# Labile Photo‐Induced Free Radical in α‐Ketoglutaric Acid: a Universal Endogenous Polarizing Agent for In Vivo Hyperpolarized ^13^C Magnetic Resonance

**DOI:** 10.1002/anie.202112982

**Published:** 2021-11-25

**Authors:** Adam P. Gaunt, Jennifer S. Lewis, Friederike Hesse, Tian Cheng, Irene Marco‐Rius, Kevin M. Brindle, Arnaud Comment

**Affiliations:** ^1^ Cancer Research UK Cambridge Institute University of Cambridge Robinson Way Cambridge CB2 0RE UK; ^2^ General Electric Healthcare Pollards Wood, Nightingales Lane Chalfont St Giles HP8 4SP UK

**Keywords:** dynamic nuclear polarization, imaging agents, keto acids, metabolism, photochemistry

## Abstract

Hyperpolarized (HP) ^13^C magnetic resonance enables non‐invasive probing of metabolism in vivo. To date, only ^13^C‐molecules hyperpolarized with persistent trityl radicals have been injected in humans. We show here that the free radical photo‐induced in alpha‐ketoglutaric acid (α‐KG) can be used to hyperpolarize photo‐inactive ^13^C‐molecules such as [1‐^13^C]lactate. α‐KG is an endogenous molecule with an exceptionally high radical yield under photo‐irradiation, up to 50 %, and its breakdown product, succinic acid, is also endogenous. This radical precursor therefore exhibits an excellent safety profile for translation to human studies. The labile nature of the radical means that no filtration is required prior to injection while also offering the opportunity to extend the ^13^C relaxation time in frozen HP ^13^C‐molecules for storage and transport. The potential for in vivo metabolic studies is demonstrated in the rat liver following the injection of a physiological dose of HP [1‐^13^C]lactate.

## Graphical abstract

α‐Ketoglutaric acid (α‐KG) has an exceptionally high free radical yield under photo‐irradiation at cryogenic temperature. This radical can be used as a universal polarizing agent to hyperpolarize ^13^C‐molecules such as [1‐^13^C]lactate, a promising alternative to pyruvate for in vivo metabolic studies by ^13^C magnetic resonance.

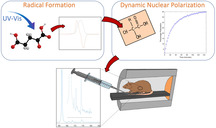

## Introduction

Metabolic imaging using hyperpolarized ^13^C magnetic resonance spectroscopic imaging (HP ^13^C MRSI) is a unique modality providing real‐time in vivo information about cellular metabolism in a non‐invasive and non‐radiative manner.^[1]^ Most studies have been performed following the injection of HP [1‐^13^C]pyruvate, the only substrate that has been translated to humans to date.^[2]^ Pyruvate not only plays a central role in intermediary metabolism, but it can also be readily polarized by dynamic nuclear polarization (DNP) when in its neat acid form and it has relatively long, and field‐insensitive liquid‐state ^13^C longitudinal relaxation times, on the order of 1 min for both the C1 and C2 carbons. Nevertheless, because of the rapid loss of hyperpolarization, the technology currently available for clinical applications requires tight synchronization of the polarization and injection of a HP [1‐^13^C]pyruvate bolus, making it a challenging procedure.

The polarization procedure can be decoupled from injection and ^13^C MR signal acquisition by replacing the persistent radical used as a polarizing agent by a labile radical that is photo‐generated in frozen neat pyruvic acid.^[3]^ This radical is not only quenched upon dissolution of the frozen pyruvic acid in water, removing the need for a filtration process and associated quality control required for clinical applications when synthetic radicals are used,^[4]^ but it can also be quenched by simply warming the frozen pyruvic acid to above 190 K. The latter can be done while maintaining the enhanced ^13^C spin polarization built up via DNP, allowing storage and transport of the polarized ^13^C‐pyruvic acid.^[5]^

Although widely used, HP [1‐^13^C]pyruvate yields metabolic data that can be difficult to interpret because of the supraphysiological dose that is injected intravenously. Recently it has been shown that high exogenous pyruvate concentrations inhibit lactate dehydrogenase (LDH) activity in cells,^[6]^ and that highly concentrated bolus injections of pyruvate in vivo inhibit or saturate pyruvate dehydrogenase (PDH) in the rat liver.^[7]^ The two alternative ^13^C‐substrates that can be injected at higher concentration to probe carbohydrate metabolism without substantially increasing their plasma level are lactate and glucose, due to their high inherent blood concentrations. HP [1‐^13^C]lactate has already been used in several preclinical applications,^[8]^ and shows promise for clinical studies. However, it has so far never been injected and observed at doses low enough to maintain physiological plasma levels. HP [U‐^2^H, U‐^13^C]glucose has been used for in vivo preclinical studies at physiological concentrations,^[9]^ but is a challenging substrate because of the short ^13^C T_1_s, on the order of 12 s in vitro and 7 s in vivo. ^13^C‐labelled glucose has been hyperpolarized using a photo‐generated radical, although the radical precursor used is an exogenous synthetic molecule (trimethylpyruvic acid) with an unknown safety profile for in vivo applications, especially as a large concentration (≥0.7 M) needs to be admixed with glucose for this purpose.^[10]^ Pyruvate could also be used to co‐polarize glucose, as was done with butyrate,^[11]^ or acetate,^[12]^ but even if not ^13^C‐labeled, it would still interfere with the metabolism being investigated. Here we propose the use of a radical photo‐generated in the endogenous molecule alpha‐ketoglutaric acid (α‐KG) as a universal endogenous polarizing agent for HP ^13^C MR. We demonstrate its efficiency by using it to prepare a HP [1‐^13^C]lactate solution that is injected at a physiological dose in rats to probe hepatic metabolism in vivo.

## Results and Discussion

**Figure 1:**
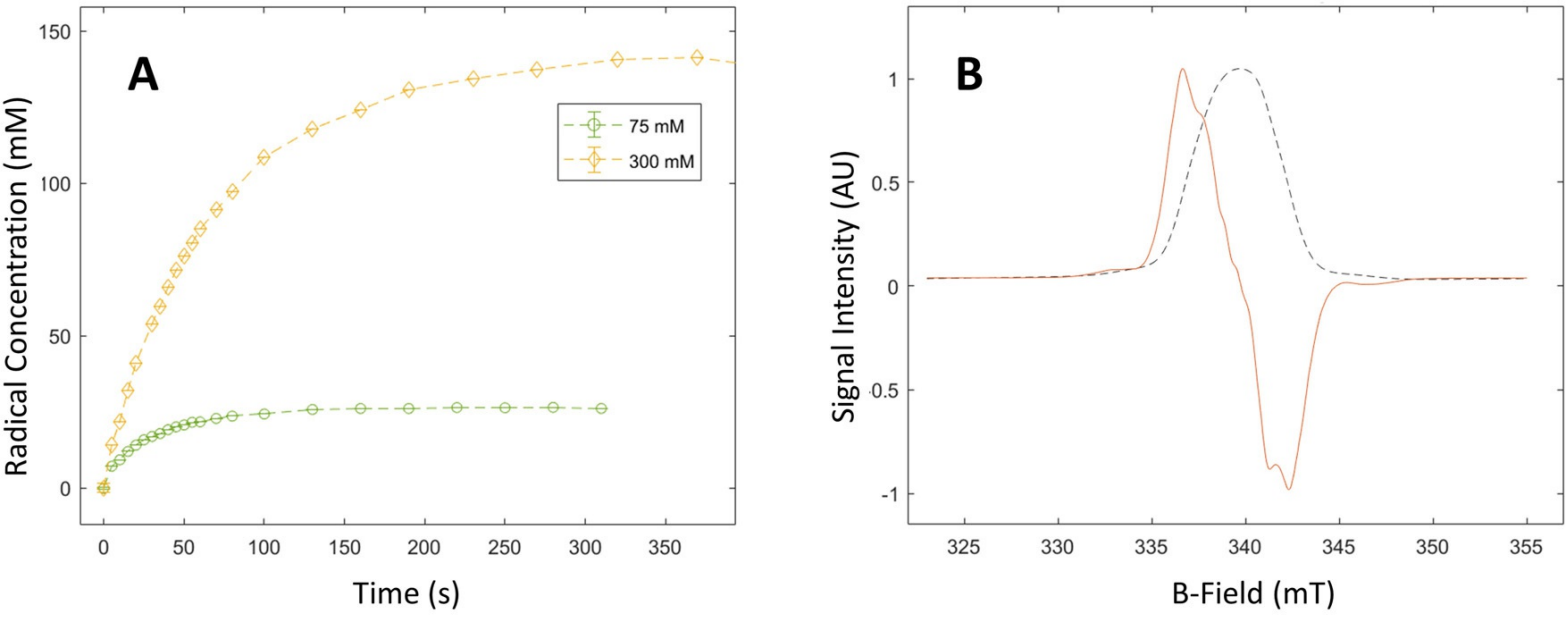
Production of photo‐induced radicals in a solution of 80:20 lactic acid:water (w:w) and α‐KG. A. Radical concentration as a function of the photo‐irradiation time for 75 mM (green circles) and 300 mM (yellow diamonds) of α‐KG. The build‐up time constant was 66 s for the sample containing 300 mM α‐KG and 31 s for the 75 mM sample. The dashed lines show the exponential fit used to extract the radical build‐up times. B. Normalized X‐band ESR dispersion and absorption spectra for the photo‐generated radical in the 300 mM α‐KG sample at the 75 s time point.

α‐KG exhibits an extraordinarily high radical conversion rate under ultraviolet‐visible (UV/Vis) light irradiation at 77 K when admixed with an aqueous lactic acid solution. The radical concentration deduced from the X‐band electron spin resonance (ESR) spectrum recorded at 77 K after 100 s of irradiation was 26±2 mM in a frozen 80:20 lactic acid:water (w:w) solution (6 μL vitrified droplet or “bead”) containing a starting concentration of 75 mM of α‐KG, corresponding to a radical yield of 35 % (Figure 1 a). When the starting concentration of α‐KG was increased to 300 mM, a radical concentration of 150±10 mM was reached after 300 s, corresponding to a 50 % conversion rate. Such a free radical concentration is however significantly greater than the optimal concentration required for DNP, and for the preparation of the samples used for HP ^13^C experiments the irradiation time for 300 mM α‐KG was shortened to 30 s in order to photo‐generate a radical concentration of 50±5 mM. The observed linewidth of the X‐band ESR spectrum was 6.57 mT (Figure 1 b).

**Figure 2:**
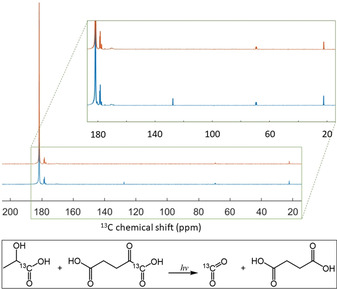
^13^C MR spectra of diluted (3:50 v:v in H_2_O) photo‐irradiated (blue) and non‐irradiated (orange) 80:20 [1‐^13^C]lactic acid:water (w:w) solution containing 300 mM [1‐^13^C]α‐KG. The [1‐^13^C]lactic acid resonance is the large peak at 183.2 ppm (natural abundance lactic acid C2 and C3 doublets are visible at 69.6 and 21 ppm, respectively). The [1‐^13^C]α‐KG resonance is at 170.8 ppm. Three peaks from lactic acid impurities were detected within the 177–180 ppm region. The inset highlights the ^13^CO_2_ peak (127.6 ppm) that was only detected in the photo‐irradiated sample.

To identify photo‐generated byproducts, photo‐irradiated and non‐photo‐irradiated frozen 80:20 [1‐^13^C]lactic acid:water (w:w) samples, containing 300 mM [1‐^13^C]α‐KG, were dissolved in water and analyzed by liquid‐state ^13^C MR spectroscopy. As expected from analogous experiments performed previously with photo‐irradiated frozen keto‐acids,[^3^, ^13^] the UV/Vis light induces the decarboxylation of α‐KG. ^13^CO_2_ was the only detectable breakdown product in the ^13^C MR spectrum of the photo‐irradiated sample. Although succinic acid is the other expected byproduct, its ^13^C MR signal could not be resolved because it is unlabeled and moreover its ^13^C resonance will be obscured by the large [1‐^13^C]lactic acid peak. However, liquid chromatography‐mass spectrometry (LC‐MS) analysis of a photo‐irradiated sample of 300 mM α‐KG in water confirmed the presence of succinic acid (see supplementary information). The concentration of ^13^CO_2_ estimated from the ^13^C MR spectrum of a 300 mM [1‐^13^C]α‐KG solution photo‐irradiated for 30 s was 50±10 mM (Figure 2), which corresponds to the maximum solubility of carbon dioxide in water at room temperature and is equivalent, as expected, to the radical concentration determined by X‐band ESR at 77 K. In HP ^13^C MR experiments, the concentration of radical breakdown products is further diluted by a factor of 200±30 post dissolution, resulting in a maximum expected concentration of 250 μM for a sample containing 50 mM of photo‐induced free radicals.

**Figure 3:**
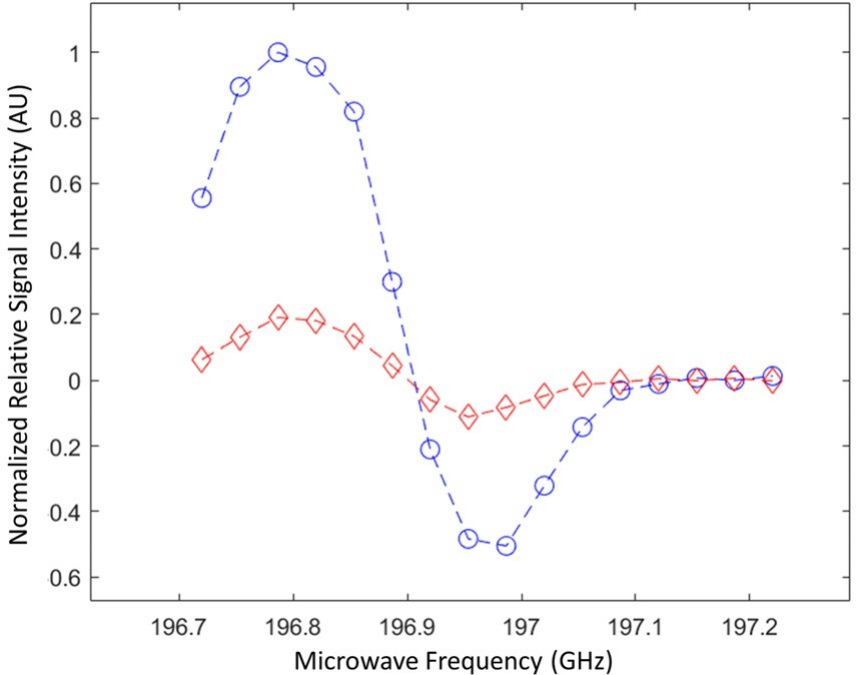
Microwave DNP spectrum of a photo‐irradiated 80:20 [1‐^13^C]lactic acid:water (w:w) solution containing 300 mM [1‐^13^C]α‐KG. The measurements were performed at 7 T and 1.35 K with (blue dots) and without (red diamonds) microwave frequency modulation. Microwave frequency was modulated with an amplitude of 52 MHz and a rate of 1.5 kHz.

**Figure 4:**
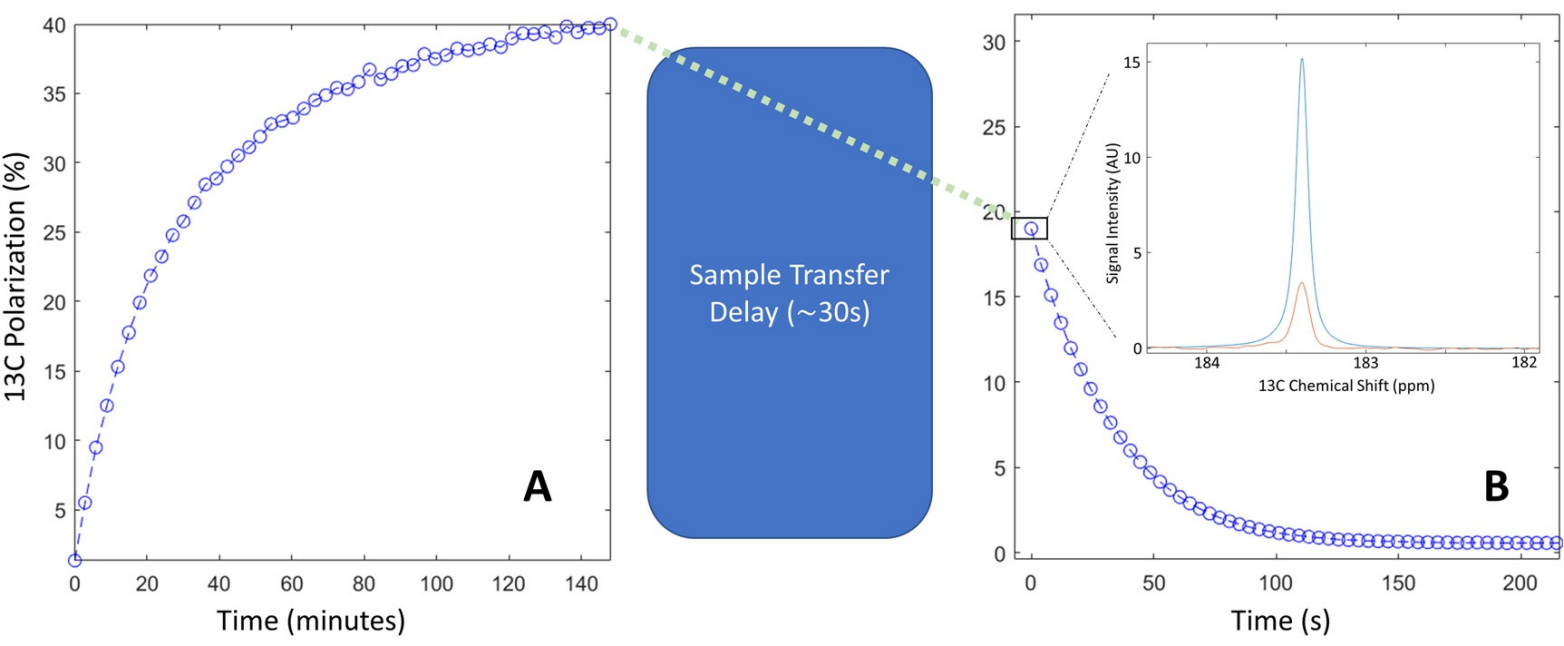
^13^C polarization measured inside the polarizer and following dissolution. A) Solid‐state ^13^C polarization build‐up recorded in a photo‐irradiated 80:20 [1‐^13^C]lactic acid:water (w:w) sample containing 300 mM [1‐^13^C]α‐KG. B) Liquid‐state ^13^C polarization measured at 14.1 T. The first HP ^13^C spectrum recorded after sample transfer is displayed in the inset together with the thermally polarized ^13^C spectrum used to determine the liquid‐state ^13^C polarization. The integrals of the two peaks are 3.0±0.1 and 1.6±0.1 for the HP and thermally polarized signals, respectively.

The performance of the radical photo‐induced in α‐KG as a polarizing agent for DNP was assessed by measuring the maximum solid‐state ^13^C polarization at 7 T and 1.35 K as a function of the microwave frequency in a photo‐irradiated frozen 80:20 [1‐^13^C]lactic acid:water (w:w) sample containing 300 mM α‐KG (Figure 3). Modulation of the microwave irradiation frequency increased the solid‐state ^13^C polarization at the optimum polarizing microwave frequency by a factor 5. The ^13^C polarization build‐up curve at the optimal microwave frequency (196.778 GHz), with a microwave output power of 25 mW, followed a mono‐exponential function with a time constant of 50±5 min (Figure 4 a). The maximum solid‐state ^13^C polarization after 3 h of microwave irradiation was 40±10 %.

The HP ^13^C MR [1‐^13^C]lactate signal measured at 30 s post dissolution was compared to the thermally polarized ^13^C MR signal and gave a liquid‐state ^13^C polarization of 19±2 % (*n*=5). (Figure 4 b). The room‐temperature longitudinal ^13^C relaxation time constant (T_1_) at 14.1 T was 36±2 s. Assuming that the ^13^C polarization during the 30 s transfer time decreased with a low‐field ^13^C T_1_ of 50 s, the calculated ^13^C polarization at the start of the dissolution process was estimated to be 35±5 %. The dissolution is driven by high pressure helium gas and, as a result, the sample degasses during the dissolution process and the residual ^13^CO_2_ concentration was undetectable.

**Figure 5:**
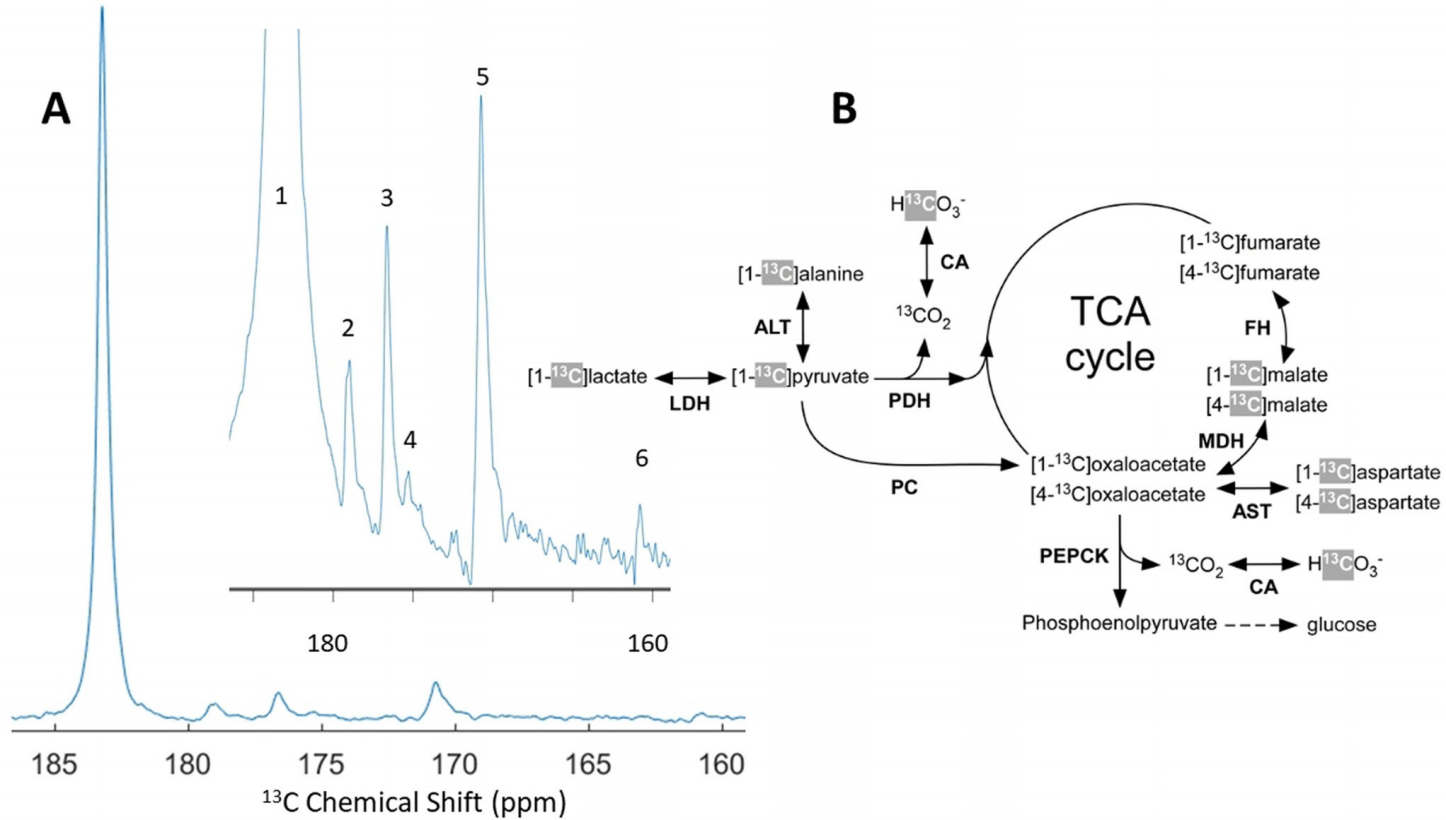
HP ^13^C MR experiments in the rat liver in vivo. A. Sum of the ^13^C spectra acquired with a ^13^C surface coil placed on top of the rat liver region between 34 and 74 s following injection of a 1 mL 42 mM [1‐^13^C]lactate bolus via the tail vein. The inset spectrum highlights the area of interest containing the lactate metabolites.^13^C signals (numbered 1 to 6) from the injected [1‐^13^C]lactate (183.7 ppm–#1) and its metabolic products ([1‐^13^C]pyruvate (171 ppm–#5), [1‐^13^C]pyruvate hydrate (179 ppm–#2), [1‐^13^C]alanine (176.7 ppm–#3), [1‐^13^C]aspartate (175.2 ppm–#4), and [^13^C]bicarbonate (161 ppm–#6) were detected in all experiments. The [1‐^3^C]malate (180.6 ppm) signal should also be present but is obscured by the shoulder of the [1‐^13^C]lactate peak. B. Metabolic pathways showing the flow of ^13^C label from [1‐^13^C]lactate into the TCA cycle and gluconeogenesis pathway in the liver. ^13^C labelling from the injected lactate is first converted to C1 of pyruvate. The ^13^C labels highlighted in grey are the ones that can be expected to be observed in the HP ^13^C spectra. Enzymes are abbreviated in bold font: lactate dehydrogenase (LDH), alanine aminotransferase (ALT), pyruvate dehydrogenase (PDH), carbonic anhydrase (CA), pyruvate carboxylase (PC), malate dehydrogenase (MDH), fumarate hydratase (FH), aspartate aminotransferase (AST), and phosphoenolpyruvate carboxykinase (PEPCK).

A series of ^13^C spectra were recorded using a ^13^C surface coil placed on top of the rat liver region every 2 s following the intravenous injection (tail vein) of a 1 mL bolus of a 42±3 mM [1‐^13^C]lactate solution (Figure 5). In addition to the resonance of the injected [1‐^13^C]lactate, the following downstream metabolites could be detected (*n*=2): [1‐^13^C]pyruvate (170.7 ppm) and [1‐^13^C]pyruvate hydrate (179 ppm), [1‐^13^C]alanine (176.7 ppm), [1‐^13^C]aspartate (175.2 ppm), [1‐^13^C]malate (180.6 ppm), and [^13^C]bicarbonate (160.8 ppm). [2‐^13^C]malate may have been present however it would be obscured by the shoulder of the lactate peak at 181.7 ppm. The calculated lactate/pyruvate and alanine/pyruvate ratios were 15±2 and 0.8±0.2 respectively.

Although other keto acids have been proposed previously as free radical precursors for in vivo HP ^13^C MR,[^11^, ^13^, ^14^] the exceptionally high radical yield together with the excellent safety profile of α‐KG makes this molecule attractive for translation to humans. The molecule indeed has been shown to be beneficial when injected i.v. at doses of up to 200 mmol h^−1^ in patients undergoing heart surgery.^[15]^ The high radical yield allows the starting concentration of α‐KG in the sample to be very low; in this study the concentration of α‐KG in the 1 mL bolus was less than 1.5 mM. In addition, the high yield decreases the UV irradiation time required to reach the target radical concentration. The slow cell uptake of α‐KG^[16]^ means that within the short time frame of a HP ^13^C MR experiment, it is not expected to alter the metabolic response to the molecule of interest, in this case lactic acid. Moreover, α‐KG and its breakdown products (CO_2_ and succinate) are endogenous molecules which would not be of any concern for injection into humans, especially at doses lower than 5 μmol kg^−1^ for α‐KG and 1 μmol kg^−1^ for succinic acid and CO_2_, as was the case in this study.

The X‐band ESR line width of the free radical photo‐induced in α‐KG is approximately 11 % narrower than that in photo‐irradiated pyruvic acid, although it is not as narrow as for some exogenous photo‐irradiated keto acids.[^10^, ^13^] This parameter, however, is less relevant for DNP at high fields, especially at 7 T as demonstrated in the present study, where microwave frequency modulation can be used to compensate for a wider microwave spectrum.^[17]^ The unusually large ^13^C polarization enhancement factor of 5 induced by microwave frequency modulation also demonstrates that other properties of the free radical photo‐induced in α‐KG, such as its spin‐lattice relaxation rate, are favorable for DNP at 7 T. The 40±10 % ^13^C polarization at the time of dissolution determined from direct solid‐state ^13^C MR measurements, and corroborated by back‐calculation from the liquid‐state ^13^C polarization measured in vitro, is comparable with the 50 % ^13^C polarization obtained in [1‐^13^C]lactic acid with trityl radicals at 5 T and 0.8 K.^[8e]^ Assuming a linear dependence of the ^13^C polarization on B_0_/T,^[18]^ the two results are indeed equivalent.

The large ^13^C polarization allowed measurement of the real‐time metabolism in vivo of a low dose of HP [1‐^13^C]lactate (0.12 mmol kg^−1^), which gives a plasma concentration of approximately 1.8±0.4 mM in a 350 g rat.^[19]^ This represents an increase in the plasma concentration of less than a factor of two as compared to basal plasma concentration, which is on the order of 2 mM in rats.^[8a]^ Therefore, the signal intensity ratio between the metabolic product [1‐^13^C]pyruvate and the injected [1‐^13^C]lactate should be close to the total (labelled and non‐labelled) pyruvate/lactate ratio if label exchange is sufficiently rapid. The lactate‐to‐pyruvate ratio of 15±2 observed in our experiments is similar to a ratio of 8–15 measured in the perfused rat liver.^[20]^ The low alanine/pyruvate ratio observed here implies that a large fraction of the labeled pyruvate is used for mitochondrial metabolism.

α‐KG was not detected in the in vivo experiments, and it is also unlikely that α‐KG has any influence on the outcome of the HP ^13^C measurements despite the fact that the estimated plasma concentration immediately after injection (60 μM) is 2.5‐fold larger than the normal physiological concentration.^[21]^ Given the exceptional yield of the α‐KG radical, its concentration could be reduced by up to a factor 4 if needed, bringing it closer to physiological levels. The concentration of succinic acid, a radical breakdown product, in the injected lactate solution was estimated to be less than 3 μM, compared to a normal plasma concentration of 17 μM.^[21]^

## Conclusion

We have demonstrated that α‐KG can be used as a free‐radical precursor for the preparation of HP ^13^C‐labelled molecules, which can be used to probe metabolism in vivo. The high levels of polarization achieved in [1‐^13^C]lactate allow injection at physiological concentrations. The high radical yield and safety profile of α‐KG and its recombination products following UV/Vis irradiation offer the possibility of translating this method to human studies. An unexplored advantage in the use of α‐KG is that the radical is labile and can be quenched in the solid state, opening up the opportunity for polarization storage and transport, as has been demonstrated with other such non‐persistent radicals.[^5^, ^22^]

## Conflict of interest

Arnaud Comment was employed by General Electric Medical Systems Inc. at the time of manuscript preparation and submission.

## Supporting information

As a service to our authors and readers, this journal provides supporting information supplied by the authors. Such materials are peer reviewed and may be re‐organized for online delivery, but are not copy‐edited or typeset. Technical support issues arising from supporting information (other than missing files) should be addressed to the authors.

Supporting Information
